# Demographics and gender-related measures in younger and older adolescents presenting to a gender service

**DOI:** 10.1007/s00787-022-02082-8

**Published:** 2022-11-12

**Authors:** Marijn Arnoldussen, Frédérique B. B. de Rooy, Annelou L. C. de Vries, Anna I. R. van der Miesen, Arne Popma, Thomas D. Steensma

**Affiliations:** 1 Department of Child and Adolescent Psychiatry, Center of Expertise On Gender Dysphoria, Amsterdam Medical Centers, Location VU, Amsterdam, The Netherlands; 2 Department of Medical Psychology, Amsterdam Medical Centers, Location VU, Amsterdam, The Netherlands

**Keywords:** Gender incongruence, Transgender, Age, Adolescents, Developmental pathways

## Abstract

Transgender adolescents may present to gender identity specialty services earlier or later in adolescence. The aim of this study was to examine whether, ‘younger’ and ‘older’ presenters could be identified in a large cohort of transgender adolescents and if differences exist between the two groups. The study sample consisted of 1487 adolescents (506 birth-assigned males, 981 birth-assigned females) referred between 2000 and 2018. The distribution of age at intake was evaluated. Demographic, diagnostic, and treatment characteristics, the Recalled Childhood Gender Identity/Gender Role Questionnaire (RCGI) to measure childhood gender nonconformity and the Body Image Scale (BIS) to measure body image were collected. Based on a stem-and-leaf plot and a histogram, two groups were identified: adolescents presenting at ≤ 13.9 years (‘younger presenters’) and adolescents presenting at 14 years or older (‘older presenters’). The sex ratio was more extreme in the group of older presenters favoring birth-assigned females (*Χ*^2^(1, *N* = 1487) = 19.69, *p* < 0.001). Furthermore, more adolescents from the younger presenting group lived with both biological parents (*Χ*^2^(1, *N* = 1427) = 24.78, *p* < 0.001), were diagnosed with gender dysphoria and started with medical gender-affirming treatment (*Χ*^2^(1, *N* = 1404) = 4.60, *p* = 0.032 and *Χ*^2^(1, *N* = 1487) = 29.16, *p* < 0.001). Younger presenters showed more gender nonconformity in childhood (*β* 0.315, *p* < 0.001, 95% CI 0.224–0.407). Older presenters were more dissatisfied with various aspects of their bodies (*p* < 0.001). The differences between older and younger presenting adolescents suggest that there may be different developmental pathways in adolescents that lead to seeking gender-affirming medical care and argues for more tailored care.

## Introduction

 In the Diagnostic and Statistical Manual of Mental Disorders 5 (DSM-5), gender dysphoria is defined as an incongruence between experienced gender identity and birth-assigned gender resulting in distress [1]. The term transgender includes people whose gender identities or gender roles differ from those usually associated with the birth-assigned gender [2].

 In the last decade, a sharp increase in adolescent-referrals to transgender services has been reported worldwide [3–5]. This development has been paralleled by an increase in research into transgender adolescents, both in adolescents from clinical and community-based samples [6–8]. Research has suggested several explanations for this striking increase in referrals [9]. A recent study from our specialized transgender service found that demographic, diagnostic, and treatment characteristics, and mental health difficulties of referred adolescents had changed little over a time period of 16 years and therefore, it has been suggested that the increase is due to the fact that gender dysphoria is more common than originally expected, with increased publicity and visibility possibly helping young people to recognize their gender incongruence and come out to their social environment. However, some are concerned that recent referrals present with qualitatively different phenomenology than earlier referrals and warn that their presentation of gender incongruence (i.e., a discrepancy between one’s gender identity and the gender one was assigned at birth) represents other mental health difficulties instead of gender incongruence [10–12]. A survey on parents of primarily birth-assigned females hypothesized what was called; ‘rapid-onset gender dysphoria’ (RODG). Characteristic for this ‘ROGD’ would be the absence of childhood gender nonconformity and the perceived suddenness of onset/presentation and it would occur mainly in birth-assigned females. Social and peer contagion and a maladaptive coping mechanism to avoid feeling strong or negative emotions were hypothesized as underlying mechanisms for this suggested subtype [11].

 Although 'ROGD' has only been recently suggested as a subtype, clinical expert papers on healthcare for transgender adolescents have since long suggested that there are different subgroups within the group of adolescents [13–15]. Some adolescents with gender incongruence present with a long history of gender nonconformity from early childhood on (pre-pubertal) but other transgender adolescents declare gender incongruence around or after puberty (peri/post-pubertal). It was usually suggested that most adolescents with a request for medical interventions have a history of early childhood-onset gender incongruence and people with late or post-pubertal gender incongruence present themselves to gender services only later in life [16, 17]. At present, still little is known about different gender identity developmental trajectories in adolescents and age of presentation [18].

A study by Sorbara et al. (2019) provided some initial thoughts on possible different gender identity developmental pathways [19]. They investigated in a cross-sectional study whether younger presenting adolescents were different compared with older presenting adolescents. In this study, the age of 15 years was chosen to divide the referrals into a younger presenting group and an older presenting group, as this ensured that the older presenting group had experienced significant pubertal development. The study showed that late pubertal stage and older age are associated with increased mental health difficulties in gender diverse adolescents, suggesting a particular vulnerability of this group and the need for tailored care. In addition, this study also found that the group of ’younger presenters’ had a significantly lower median age of recognition of gender incongruence [19]. Another study comparing baseline mental health, well-being, and gender-specific experiences between a cohort of youth receiving puberty-inhibiting (GnRHa) treatment and a cohort of youth in whom the vast majority (93%) received gender-affirming hormones (GAH) directly without initial GnRHa treatment because of their age found that youth from the GnRHa cohort recognized that their gender was incongruent with their birth-assigned gender at approximately 4 years younger than youth from the GAH cohort, and accessed gender-affirming medical treatment (GAMT) earlier in their development. In addition, mental health, well-being, and body image was found to be better in the GnRHa cohort compared with the GAH cohort [20]. The findings from these studies suggest that there might be different ages of recognition of and/or onset for feelings of gender incongruence, and thereby, possibly different developmental pathways.

Because there may be different developmental pathways and/or different ages of declaration of gender incongruence, presumably not everyone will benefit from the same standardized medical treatment, but instead it is important to provide an individualized approach [21]. However, in transgender adolescent care, care is often offered in the form of a protocol [22, 23]. Although protocols allow for some tailored care, the use of protocols does imply that the same general guidance is offered to each adolescent. Gaining more insight into the heterogeneity of adolescents presenting to specialized transgender care services may help to inform clinicians and offer more individualized approaches.

In summary, at present, adolescents visiting gender identity specialty services are often treated according to the same guidelines that may not take into account the differences that might exist regarding age of presentation and experiences of gender incongruence. Therefore, the aim of this study was to gain better insight into the age distribution of the adolescents who presented at our specialized transgender service over the years. We wanted to examine whether, like other studies have shown, ‘younger’ and ‘older’ presenters can be identified and if so, if differences existed with regard to demographic, diagnostic, and treatment characteristics, recalled childhood gender nonconformity, and body image.

## Methods

### Participants and procedure

The study sample consisted of 1487 adolescents (range 8.9–18.4 years) who were consecutively referred to the Center of Expertise on Gender Dysphoria in Amsterdam (CEDG) between 2000 and 2018. The term "adolescents" was used for all youth who had been referred because of their desire for GAMT, which meant that youth as young as age 9–10 were included because puberty may start at that age.

As part of the standard procedure in the assessment session at the CEDG, sociodemographic characteristics and various questionnaires on general and psychological functioning as well as on gender identity (including the RCGI and the BIS), were collected upon first assessment (for an overview see de Vries et al. 2014 [24]). The data whether an adolescent fulfilled the criteria for a gender dysphoria diagnosis and whether they started with GAMT were collected after the assessment sessions of the adolescents.

### Measures

#### Demographics

The following demographic measures were included: age at time of first assessment, birth-assigned gender, parents’ marital status, parents’ educational level, total IQ of the adolescents, gender dysphoria diagnosis, and start with medical treatment. Because the gender identities of these adolescents were not yet within our knowledge at the time of intake, this study refers to birth-assigned males and birth-assigned females.

Parents’ marital status was classified as either living with both biological parents or other (e.g., divorced, living in a group home). Parents’ educational level was categorized as either “vocational educated” or “higher vocational educated or academic educated”. Vocational education prepares for work in a specific trade or craft, whereas higher vocational or academic education focuses on theory and knowledge.

Total IQ was assessed using the Dutch version of the Wechsler Intelligence Scale for Children for adolescents aged 15 years or younger [25, 26], and the Wechsler Adult Intelligence Scale for adolescents aged 16 and over [27].

#### Diagnostic and treatment characteristics

Up to 2015, the diagnosis Gender Identity Disorder according to the DSM-IV-TR was used [28]. With the release of the DSM-5, this was changed to the diagnosis gender dysphoria according to the DSM-5 criteria [1]. For all adolescents, it was coded whether they fulfilled the criteria for a gender dysphoria diagnosis (DSM-IV-TR and DSM-5). It was also coded whether they started with GAMT in the form of puberty blockers and/or GAH.

#### Recalled childhood gender nonconformity

The Recalled Childhood Gender Identity/Gender Role Questionnaire (RCGI) is a 23-item self-completed questionnaire containing questions on recalled gender experience and behavior from childhood. Items are rated on a 5-point response scale ranging from 1 to 5. It measures typically gender conforming/gender nonconforming preferences and behavior, as well as relative closeness to either parent. A lower score reflects more gender nonconforming behavior in childhood [29]. Factor analysis by Zucker et al. identified two factors: gender identity and behavior and parent–child relations. Only the factor on gender identity and behavior was used for this study. Zucker et al. also reported that tests of discriminant validity indicated the potential to identify significant variation in factor scores between groups.

#### Body image

The Body Image Scale (BIS) measures satisfaction with 30 body features on a 5-point Likert scale ranging from very satisfied (1) to very dissatisfied (5)**.** Higher scores represent higher degrees of body dissatisfaction [30]. Lindgren and Pauly suggested a subscale analysis of the BIS, using three different subscales: 1) primary sex characteristics, 2) secondary sex characteristics, and 3) neutral characteristics. However, through these subscales, it is not possible to compare differences per body area between age groups. For this reason, an alternative item clustering was chosen as previously applied by van der Grift et al. [31, 32]. The following subscales were used: (1) social and hair items, (2) head and neck items, (3) muscularity and posture, (4) hip region, (5) chest region, and (6) genitals. The internal consistence per scale of the BIS used in our study was considered good (all Cronbach's alpha were > 0.80, only for the scale 'head and neck items' the Cronbach's alpha was 0.76).

### Statistical analyses

All data analyses were performed using SPSS version 26. A significance level of *p* < 0.05 (two tailed) was used. The distribution of age at intake was evaluated on an individual year-by-year basis from 2000 to 2018. A stem-and-leaf plot and a histogram were made to examine whether different groups could be identified in the total cohort of adolescents. First, chi-square and independent *t* tests were used to identify whether demographic characteristics differed between age of presentation. To examine associations between age groups on the RCGI and BIS questionnaires, linear regression analyses were performed. All demographic variables were checked for confounding and parents’ marital status and treatment status added as covariates in all analyses. Birth-assigned gender was included to examine both confounding and effect modification.

## Results

### Age distribution

**Fig. 1:**
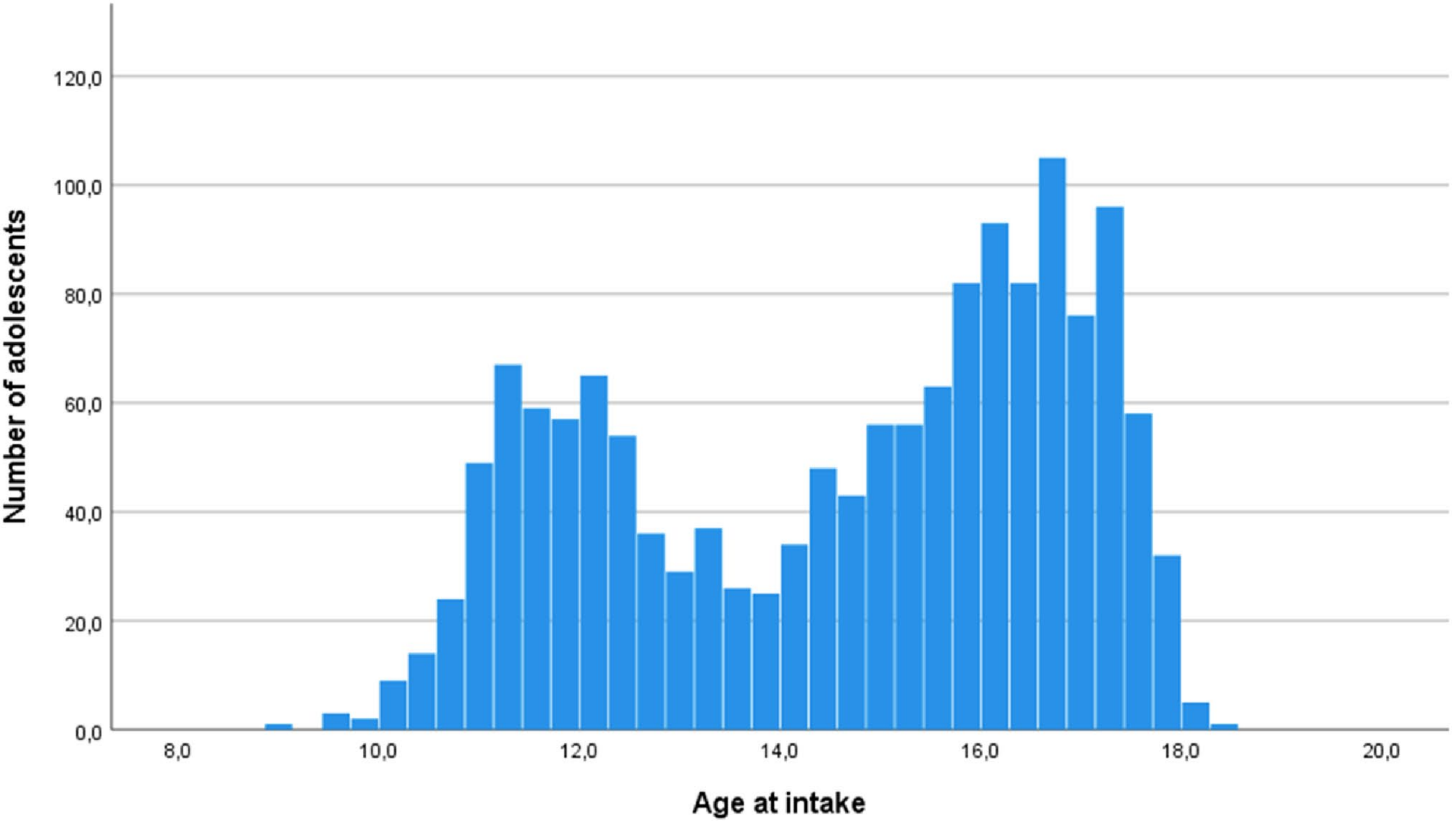
Age at intake of adolescents who were referred between 2000 and 2018

The distribution of age on an individual year-by-year basis from 2000 to 2018 was examined. It was observed that the age distribution of referrals over the years was not evenly distributed, but consistently showed the highest number of referrals around the age of 11/12 years and the age of 16/17 years within each year, as shown in Fig. 1. Based on a stem-and-leaf plot and a histogram, two groups were identified within the total cohort of 1487 adolescents based on a median split. Adolescents who were 13.9 years or younger at assessment were coded as ‘younger presenter’ (median age 11.95 years) and adolescents who were assessed at 14.0 years or older were coded as ‘older presenter’ (median age 16.25 years).

### Demographic, diagnostic, and treatment characteristics

**Fig. 2:**
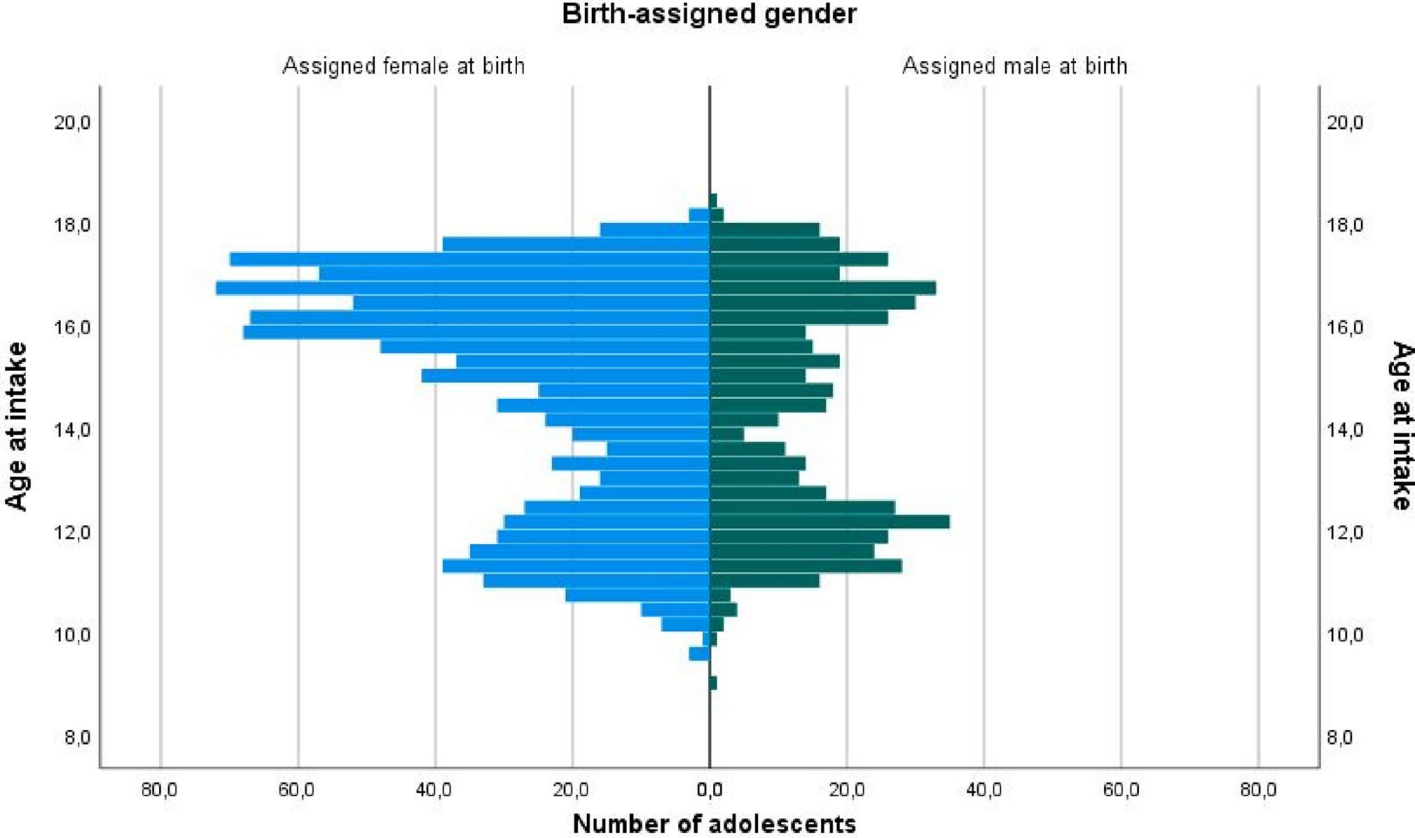
Age at intake of adolescents by birth-assigned gender

The demographic characteristics of the younger and older presenters are shown in Table 1. Several differences between younger and older presenters emerged. First, the sex ratio was more skewed in the older presenting group. Whereas in younger presenters the sex ratio was 1:1.43 favoring birth-assigned females, in older presenters it was 1:2.35 (Fig. 2). Furthermore, significantly more adolescents from the younger presenting group lived with both their biological parents. Furthermore, significantly more adolescents from the younger presenting group were diagnosed with gender dysphoria and significantly more started with medical treatment as compared with the older presenting group. The parents’ educational level and the adolescent’s full-scale IQ did not differ significantly between the younger and older presenters.

**Table 1:** Demographic, diagnostic, and treatment characteristics of ‘Younger Presenters’ and ‘Older Presenters’

Demographic variables	Younger Presenters *N* = 552	Older Presenters *N* = 935	
Age at assessment in years, *M (SD)*	12.0 (0.94) 8.9–13.9	16.2 (1.03) 14.0–18.4	
Birth-assigned gender, *N (%)*			
Assigned males at birth	227 (41.1%)	279 (29.8%)	Χ^2^(1, *N* = 1487) = 19.69, *p* < 0.001
Assigned females at birth	325 (58.9%)	656 (70.2%)	
Parents’ marital status, *N (%)*			
Living with both biological parents	352 (63.8%)	462 (49.4%)	Χ^2^(1, *N* = 1427) = 24.78, *p* < 0.001
Other	186 (33.7%)	427 (45.7%)	
Unknown	14 (2.5%)	46 (4.9%)	
Parents’ educational level, *N (%)*			
Vocational educated	251 (45.5%)	363 (38.8%)	Χ^2^(1, *N* = 1357) = 2.76, *p* = 0.097
Higher vocational or academic educated	271 (49.1%)	472 (50.5%)	
Unknown	30 (5.4%)	100 (10.7%)	
Full-scale IQ, *M (SD)*	99.53 (15.32)	99.33 (16.21)	*t*(1262) = 0.217, *p* = 0.828
Diagnosis, *N (%)*			
Gender dysphoria diagnosis	490 (88.8%)	754 (80.6%)	Χ^2^(1, *N* = 1404) = 4.60, *p* = 0.032
No gender dysphoria diagnosis	49 (8.9%)	111 (11.9%)	
Unknown	13 (2.4%)	70 (7.5%)	
Treatment			
Did start with medical treatment	470 (85.1%)	683 (73%)	Χ^2^(1, *N* = 1487) = 29.16, *p* < 0.001
Did not start with medical treatment	82 (14.9%)	252 (27%)	

*M* = mean, *SD* = standard deviation

Because the sex ratio was significantly different in the younger and older presenting group, in the following sections, the analyses were stratified for birth-assigned gender.

### Recalled childhood gender nonconformity

Table 2 and Table 3 show the mean scores and standard deviations of younger presenters and older presenters on the RCGI in birth-assigned males and birth-assigned females. Linear regression with the RCGI as the outcome variable showed that the younger presenters had a significantly lower score on the RCGI compared with older presenters when controlled for parents’ marital status and treatment status in assigned males at birth (*β *0.485, *p* < 0.001, 95% CI 0.347–0.624; *R*^2^ 0.278), meaning more extreme childhood gender nonconformity was present. A similar linear regression in birth-assigned females also showed that the younger presenters had a significantly lower score on the RCGI compared with older presenters when controlled for parents’ marital status and treatment status (*β* 0.315, *p* < 0.001, 95% CI 0.224–0.407; *R*^2^ 0.127).

**Table 2:**
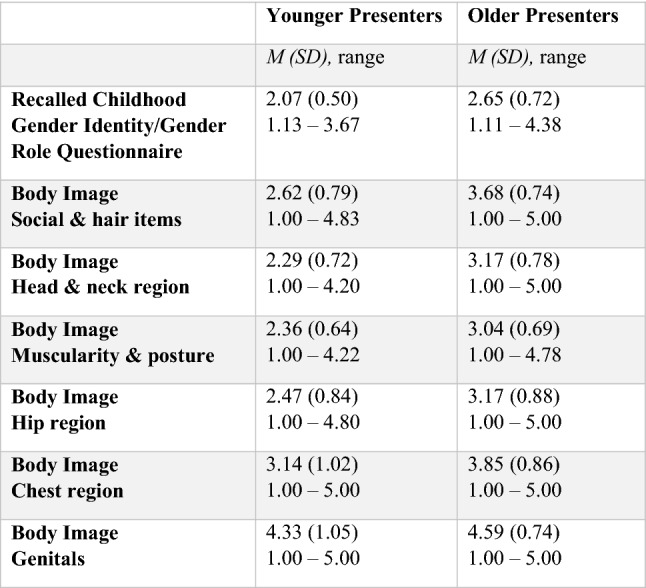
Mean scores on the recalled childhood gender identity/gender role questionnaire and body image scale of ‘younger presenters’ and ‘older presenters’ in birth-assigned males

*M* = mean, *SD* = standard deviation

**Table 3:**
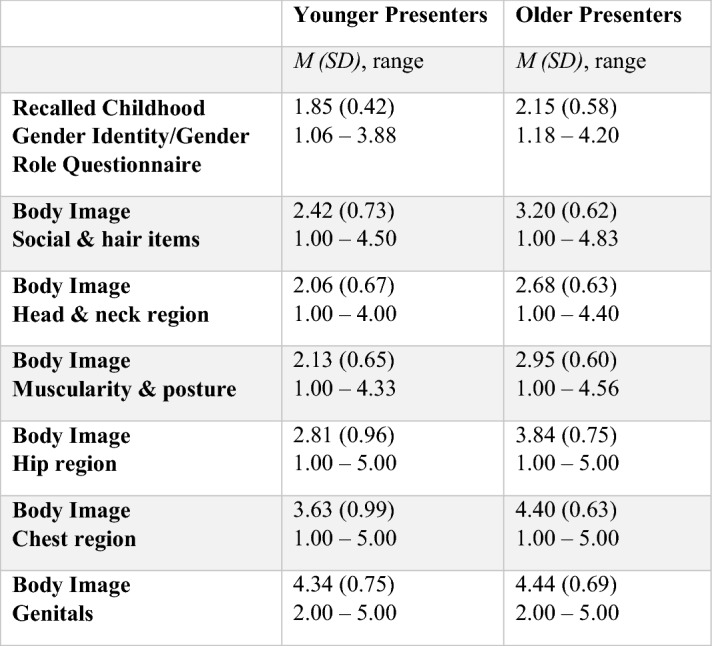
Mean scores on the recalled childhood gender identity/gender role questionnaire and body image scale of ‘younger presenters’ and ‘older presenters’ in birth-assigned females

*M* = mean, *SD* = standard deviation

### Body image

#### Social and hair items

Table 2 and Table 3 show the mean scores and standard deviations of younger presenters and older presenters on the different subscales of the BIS in birth-assigned males (Table 2) and birth-assigned females (Table 3).

In birth-assigned males, a regression analysis showed that older presenters had a significantly higher score compared with younger presenters when controlled for parents’ marital status and treatment status (*β* 1.061, *p* < 0.001, 95% CI 0.896–1.226; *R*^2^ 0.320), indicating more dissatisfaction with their appearance, body hair, body movement, facial hair, hair and voice. In birth-assigned females, older presenters also had a significantly higher score compared with younger presenters when controlled for parents’ marital status and treatment status (*β* 0.812, *p* < 0.001, 95% CI 0.704–0.919; *R*^2^ 0.236).

#### Head and neck items

In birth-assigned males, it was found that older presenters had a significantly higher score indicating more dissatisfaction on the subscale ‘head and neck items’ when controlled for parents’ marital status and treatment status (*β* 0.908, *p* < 0.001, 95% CI 0.746–1.070; *R*^2^ 0.256) compared with the younger presenters. Older presenters also had a significant higher score indicating more dissatisfaction, compared with younger presenters in birth-assigned females (*β* 0.639, *p* < 0.001, 95% CI 0.534–0.743; *R*^2^ 0.167).

#### Muscularity and posture items

In birth-assigned males, it was found that older presenters had a significantly higher score, so were more dissatisfied, on the subscale ‘muscularity and posture items’ compared with younger presenters when controlled for parents’ marital status and treatment status (*β* 0.684, *p* < 0.001, 95% CI 0.540–0.827; *R*^2^ 0.210). Older presenters also had a significant higher score, meaning more dissatisfaction, compared with younger presenters in birth-assigned females (*β* 0.839, *p* < 0.001, 95% CI 0.739–0.940; *R*^2^ 0.273).

#### Hip region items

In birth-assigned males, older presenters scored significantly higher compared with younger presenters when controlled for parents’ marital status and treatment status (*β* 0.697, *p* < 0.001, 95% CI 0.512–0.882; *R*^2^ 0.147) meaning they were more dissatisfied with the aspects of their bodies from the ‘hip region’. In birth-assigned females, older presenters also had a significantly higher score compared with younger presenters when controlled for parents’ marital status and treatment status (*β* 1.075, *p* < 0.001, 95% CI 0.943–1.207; *R*^2^ 0.264).

#### Chest region items

 In birth-assigned males, older presenters had a significantly higher score compared with younger presenters when controlled for parents’ marital status and treatment status (*β* 0.780, *p* < 0.001, 95% CI 0.581–0.979; *R*^2^ 0.142), meaning more dissatisfaction with their chest region. Older presenters also had a significant higher score compared with younger presenters in birth-assigned females (*β* 0.804, *p* < 0.001, 95% CI 0.683–0.925; *R*^2^ 0.205).

#### Genitals

In birth-assigned males, older presenters had a significantly higher score compared with younger presenters when controlled for parents’ marital status and treatment status (*β* 0.422, *p* < 0.001, 95% CI 0.239–0.606; *R*^2^ 0.118), indicating more dissatisfaction with their genitals. In birth-assigned females, no significant difference was found between younger and older presenters (*β* 0.084, *p* 0.162, 95% CI – 0.034–0.203; *R*^2^ 0.014).

## Discussion

The present study showed that over almost two decades, the distribution of the age of clinic referred transgender adolescents was not evenly distributed, but was distributed in a younger group presenting around the age of 11/12 years (median age 11.95 years) and an older group presenting around the age of 16/17 years (median age 16.25 years). Relatively more adolescents belonged to the older presenting group. This observation is comparable to other studies [19, 20], but this was the first study that determined the cut off between both groups at age 13.9 based on observation of the data, whereas other studies split the groups based on assumed puberty staging or by dividing the group based on start with puberty blockers (mean age 11.2) or GAH (mean age 16) [19, 20]. Like the other studies, the present study also revealed several differences between the younger and the older presenting group. First, both groups had more birth-assigned females, but in the younger group, the sex ratio was 1:1.43 favoring birth-assigned females, whereas in older presenters this was 1:2.35. Our findings of a different gender ratio in younger presenting youth compared to older presenting youth might suggest that different developmental pathways may exist for birth-assigned males compared to birth-assigned females, which deserves further study. In addition, a larger part of the younger presenters lived with both biological parents, whereas the older presenters more often came from divorced families or other living circumstances. Of notice, younger adolescents more frequently were diagnosed with gender dysphoria and started with GAMT. Further, younger presenters showed higher levels of gender nonconformity in childhood. Finally, older presenters were more dissatisfied with most aspects of their bodies.

The present study showed that younger and older presenting youth differed in various demographic aspects. One was that there were relatively more birth-assigned females in the older presenting group. This is of interest and might explain that the ‘shift’ in sex ratio and the overrepresentation of birth-assigned females that was observed in several other studies, concerns, for a fairly large part, the older presenters [7, 33]. One hypothesis for this overrepresentation is that it is more accepted for birth-assigned females to present themselves in their preferred gender compared with birth-assigned males [34, 35].

It is likely that not only the age at which adolescents discover their gender incongruence affects the time point at which adolescents are referred to a gender service. The age at which young persons are ready to be open about their identity probably plays a role, as well as how the social environment reacts [36]. It is notable that there are significantly more adolescents in the older presenting group who do not live with both biological parents. It is possible that it was more difficult and took more time for the parents of these adolescents to get the same perspective on how to best help their child which resulted in a more delayed reference to a gender service. So, older presenting youth may have lacked the support and help of parents that younger adolescents depend upon to be able to come out and be referred to a specialized gender service. However, it could also be that the adolescents in the older presenting group were less likely to live with both biological parents because more time has passed for these adolescents in which their family situation could have changed compared with the younger presenting group. Finally, one hypothesis could be that divorce of parents contributes to more mental health difficulties which may have interfered with a referral to a gender service.

The study of Sorbara et al. showed that older age and late pubertal stage are associated with worse mental health among gender incongruent youth presenting to a transgender service [19]. And although the study by Sorbara et al. only included adolescents who had a gender dysphoria diagnosis, it could be that the result that older age is associated with more mental health difficulties also applies for adolescents who may not (yet) have a gender dysphoria diagnosis but are seeking GAMT. It may be that co-existing mental health difficulties in older referrals are related to a less unequivocal gender identity exploration in which relatively more adolescents follow a path of which GAMT is not a part. This could be a reason that in the older presenting group fewer individuals received a gender dysphoria diagnosis and fewer went on with GAMT. Apparently, of the older referrals, fewer adolescents fulfilled the criteria of a gender dysphoria diagnosis, no indication for GAMT could be made during the exploratory psychological trajectory, or the adolescents refrained from a medical gender-affirming trajectory. It is important that future research focuses on this.

Our finding that there are two peaks in the age distribution of referrals and the differences in demographic characteristics between the younger and older presenting group could indicate that there are different developmental trajectories leading to gender incongruence in adolescence and referral for early (in contrast to adulthood) GAMT. Although the RCGI scores of adolescents from both the younger presenting group and the older presenting group were, on average, relatively low, indicating a high level of gender nonconformity during childhood, the mean RCGI score of adolescents from the younger presenting group was significantly lower in the current study. So during pre-pubertal childhood, the younger presenters showed stronger preference for (stereotypically) gender nonconforming toys and playmates and had a more gender nonconforming appearance compared with older presenters. It might well be that this more extreme gender nonconformity led parents and their children to seek GAMT at younger ages. In contrast, in the older presenting group, childhood gender nonconformity was on average less extreme, so there was probably less reason to seek early help from gender specialists. This group possibly needed more time to realize that their gender identity did not align with their birth-assigned gender and GAMT was desired. The physical changes due to puberty probably might have been essential in this, as our results demonstrate that adolescents in the older presenting group showed more body dissatisfaction compared with the adolescents in the younger presenting group. Furthermore, peer experiences might also play a role as we know from research on developmental pathways in pre-pubertal gender nonconformity [37]; because adolescents encounter more diverse people around puberty, when they start high school, adolescents from the older presenting group may have been in a better position to explore the full range of gender diversity and to figure out which identity fitted them best. Finally, the adolescents in the older presenting group may have lacked the family support that some younger presenting adolescents get [36]. The fact that a larger percentage of these adolescents came from divorced families or other living circumstances may have made it more challenging to seek appropriate care.

Although the results of the present study suggest that there may be different developmental in adolescents that lead to seeking gender-affirming medical care, our data do not allow us to conclude whether or not this suggested ‘ROGD’ subtype exists. Our results show that there was gender nonconformity in childhood in older presenters, although less extreme than in the younger presenting group, which speaks against this suggested subtype. However, we did not evaluate other hypothesized factors that would be associated with ‘ROGD’, such as mental health difficulties. Furthermore, we did not examine how gradual or sudden the onset was. A Canadian study recently examined whether they could identify the phenomenon of 'ROGD' in their clinical population (*N* = 173). They concluded that there was no 'ROGD' because the vast majority (68–86%) did not have 'recent gender knowledge' (realized their gender was different from what other people called them) and because those who did have 'recent gender knowledge' showed relatively less anxiety severity/impairment [38]. In response to this study, Littman pointed out that Bauer et al. had not used the correct definition of ‘ROGD’ because, ‘ROGD’ would not be related to having a short history of gender incongruence, but to not having gender incongruence before puberty [39]. More studies using both self and parent report measures would be needed to gain better insight in the existence of the ‘ROGD’ subtype.

This study has several clinical implications. The differences in demographic, diagnostic, and treatment characteristics, childhood gender nonconformity, and body image among adolescents from the older and younger presenting group argues for more tailored care. To ensure that each adolescent receives the treatment that best suits them, it is important to thoroughly explore all aspects of gender and general functioning with all adolescents before making decisions about further treatment [40]. The conclusion of a previous study that gender-affirming treatment earlier in life may have benefits is not necessarily founded for everyone [20]. Despite the availability of puberty blockers in the Netherlands since 2000, the largest proportion of adolescents are older before being referred to a gender service, and the majority still comes in adulthood [4]. This may be due to social or environmental factors but could also be due to intrapersonal factors.

Our results should be viewed in light of some limitations. To begin with, in this study we did not examine exactly when and how gender incongruence emerged in adolescents. The measurement instrument for gender nonconformity in childhood is also retrospective, which could possibly result in recall bias. In addition, we did not measure whether adolescents received social support although this may be important for whether or not they were referred to a gender service at an early age. Besides, this study did not evaluate mental health difficulties and whether they differed between younger and older presenters. Furthermore, it was not tracked whether participants identified outside the binary spectrum. Another limitation of this study is that it is a cross-sectional design. The younger presenting group includes different individuals than the older presenting group and, therefore, it is unknown what the effect of age is within a person and no conclusion can be drawn with regard to causal or time-related pathways. Finally, the adolescents in this study are part of a clinical sample. Therefore, we do not know if these findings can be generalized to transgender adolescents who do not enroll in a clinic or present to different gender identity specialty services around the world.

## Conclusion

Our study showed that age distribution of adolescents who present at the Amsterdam gender service shows two peaks with a younger median age of 11.95 years and an older median age of 16.25 years. The differences exist between the younger and older presenters in terms of demographic, diagnostic, and treatment characteristics, gender nonconformity in childhood, and body image, suggesting that there may be different developmental pathways in adolescents that lead to seeking gender-affirming medical care. The fact that gender incongruent adolescents present at different ages with different characteristics calls for more research to understand the differences within the population and an individualized approach in the care and treatment of transgender adolescents.
